# Construction and Characterization of Highly Infectious Full-Length Molecular Clones of a HIV-1 CRF07_BC Isolate from Xinjiang, China

**DOI:** 10.1371/journal.pone.0079177

**Published:** 2013-11-18

**Authors:** Zheng Wang, Kunxue Hong, Jing Zhang, Lei Zhang, Dan Li, Li Ren, Hua Liang, Yiming Shao

**Affiliations:** 1 State Key Laboratory for Infectious Disease Prevention and Control, Division of Research of Virology and Immunology, National Center for AIDS/STD Control and Prevention, China CDC, Beijing, China; 2 Collaborative Innovation Center for Diagnosis and Treatment of Infectious Diseases, Hangzhou, China; Shanghai Medical College, Fudan University, China

## Abstract

Among the various subtypes of the M group of human immunodeficiency virus type 1 (HIV-1), clade CRF07_BC is the most prevalent in China. To date, no strong replicable CRF07_BC infectious clone has been constructed. Here we report on the construction and characterization of highly replicable infectious molecular clones from the isolate XJDC6291 of this HIV-1 subtype. Four full-length clones pXJDC2-7, pXJDC3-7, pXJDC2-6 and pXJDC3-6 were successfully produced, but only pXJDC2-7 presented detectable infectivity and replication capability. To improve the replication capability of pXJDC2-7, a 4.8 kb region spanning from the *pol* Integrase to *nef* gene of the clone was replaced by PCR products of the corresponding fragments from the original isolate XJDC6291, which produced two clones pXJDC13 and pXJDC17 that exhibited strong replication capability. The viral stocks obtained by pXJDC-13 and pXJDC-17 transfection into 293T cells replicated efficiently in human PBMCs, human primary CD4^+^ T cells and displayed CCR5 tropism. Sequence alignment between pXJDC13, pXJDC17 and pXJDC2-7 suggested that polymorphisms in the V1V2 region may influence infectivity, and reverse genetic experiment showed that V1V2 polymorphisms may influence the infectivity of the clones but did not affect the replication capability at a significant level. pXJDC13 and pXJDC17 displayed strong replication capability and are the first full-length infectious clones of HIV-1 CRF07_BC clade in the world. The availability of CRF07_BC infectious clones provides a useful tool for a wide range of studies, including antiretroviral drug and vaccine research as related to this HIV subtype.

## Introduction

The CRF07_BC and CRF08_BC clades of HIV-1 are products of recombination between subtype B′ and subtype C viruses [1], [2] and are the dominant circulating strains in China [3]. The majority of the CRF07_BC genome is derived from subtype C, while the internal portion of its *gag*, *pol*, *env*, and *nef* genes and LTRs, as well as the first exon of the *tat* gene, were derived from subtype B (Thai- B). Although CRF07_BC is often recognized as being responsible for the rapidly expanding HIV-1epidemic in China [4], to date there are only19 published CRF07_BC near full-length genomic sequences and no full-length infectious clone. The pathogenesis and recombination mechanisms of this clade are yet to be clarified, and the virulent factors and genetic basis for the diversity of biological properties need to be studied. Thus, a full-length replication competent molecular clone specific for the CRF07_BC would serve as a valuable tool for such studies.

The construction of an infectious clone is currently a time-consuming and laborious process, and how to produce an infectious clone quickly is still a challenge. After the first HIV-1 B infectious clone NL4-3, a chimerical clone from the integrated NY5 and LAV proviruses [5] was successfully constructed, several HIV infectious clones of different subtypes were produced successfully, including the Thai-B [6], [7], CRF01_AE [8], [9], CRF08_BC [10], C [11],and D [12] clones. We previously also reported a chimeric infectious clone of CRF07_BC with weak replication ability in peripheral blood mononuclear cells (PBMCs), in which the complete 5′ and 3′ LTR as well as the partial *nef* gene were derived from the pNL4-3 [13].

In this study, we generated two highly-infectious molecular clones from the HIV-1 CRF07_BC isolate XJDC6291. The clone-derived viruses could replicate efficiently in the human PBMCs and used CCR5 as coreceptor to enter the cell, and the availability of these highly-infectious molecular clones will facilitate studies on the virulence and recombination mechanism of CRF07_BC strains circulating in China. Meanwhile, our results also extend the notion that characteristics of the V1V2 region have an effect on the infectivity of HIV but only have a slight influence on replication capability.

## Materials and Methods

### Construction of Full-length Molecular Clones from XJDC6291 Isolate

**Figure 1:**
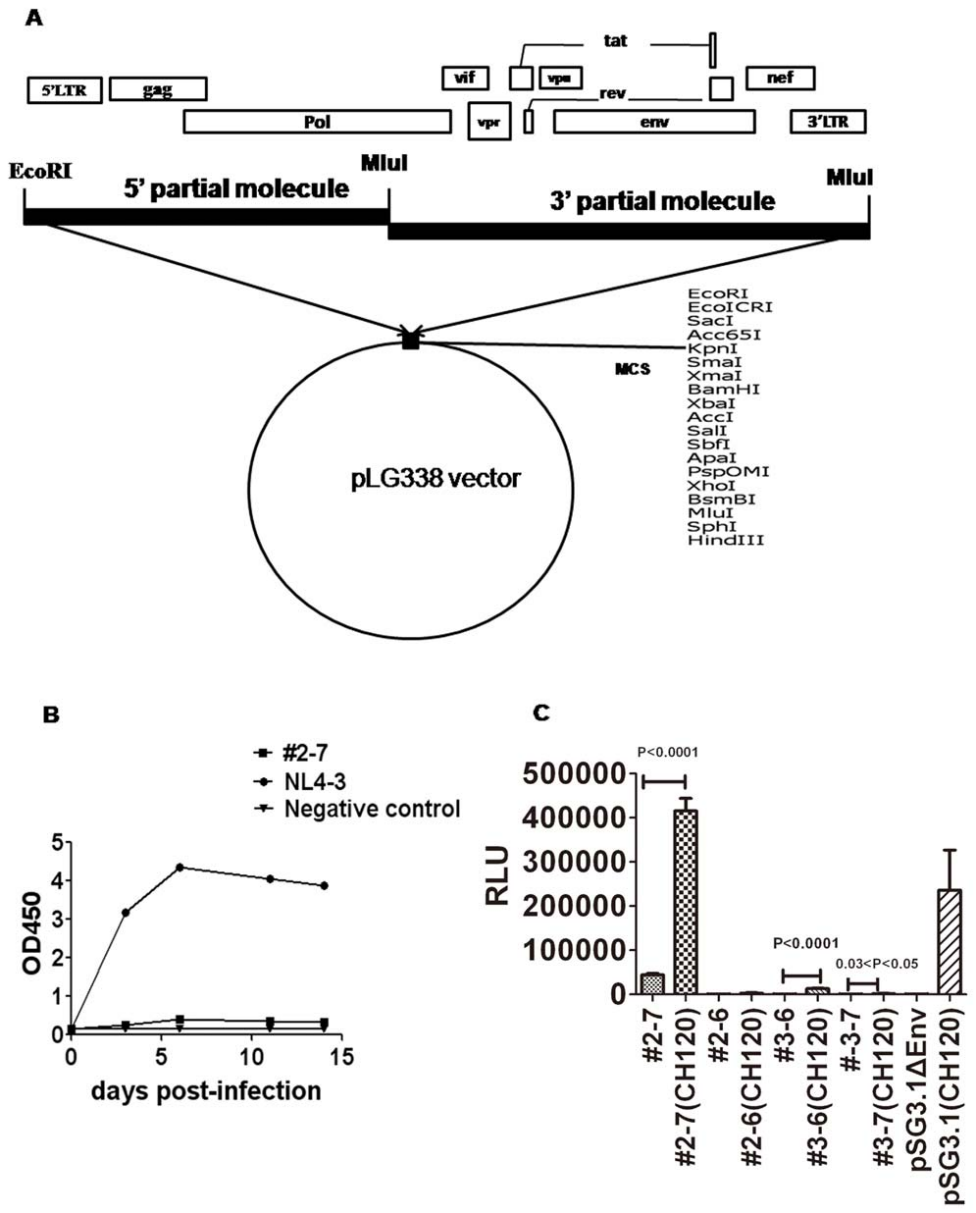
Generation of full-length molecular clones. (A) Diagram of the full-length molecular cloning method. (B) Replication capability of full-length clone pXJDC2-7 on human PBMCs. pNL4-3 derived virus was used as the positive control. Viral replication was monitored by quantifying OD value in the culture medium at defined time points using a commercial p24 antigen-capture ELISA. (C) Viral infectivity produced from co-transfection of clade BC envelope expression plasmid CH120.6 and either the full-length molecular clone. pSG3 ΔEnv HIV-1 backbone plasmid was used as positive control. The data are shown as the mean relative light unit (RLU) with standard deviation from four independent experiments.

Isolation of the HIV-1 primary isolate XJDC6291 has been described previously [14] from a 35 year old male infected individual in Xinjiang Uyghur Autonomous Region who was infected by injection drug use. The viral isolate was propagated in fresh IL-2 stimulated PBMCs, and proviral DNA was extracted using the Qiagen blood DNA kit (Qiagen, Hilden, Germany) from infected PBMCs. Specific primers were designed to amplify the full-length HIV DNA provirus (Table 1). The two partial molecules of the entire XJDC6291 genome were amplified with the Platinum Taq DNA polymerase High Fidelity kit (Invitrogen, Carlsberg, USA). For the 5′ partial molecule, primers U-LTR5-BC and Rev11 were applied for the first round PCR, and U-LTR5-BC was paired with D-Mlu-BC for the 2nd round PCR. To amplify the 3′ partial molecule, 1st round PCR primers (U-MluI-1-BC, D-MluI-AE+BC) and the 2nd round PCR primers (U-MluI-2-BC, D-MluI-AE+BC) were used for the semi-nested PCR reaction. The PCR product was purified and cloned into the low copy plasmid pLG338 sequentially using unique restriction enzyme sites to construct full-length molecular clones; for the list and locations of restriction sites, see Fig. 1.A.

**Table 1:** Primers for constructing infectious clones.

Primer	Sequence (5′-3′)	Position in HxB2
U-LTR5-BC	GCGCCGAATTCTGGAAGGGTTAATTTACTCTAAGA	1–24
Rev11	ATCATCACCTGCCATCTGTTTTCCAT	5041–5066
D-Mlu-BC	AAAATACGCGTCCCCCACATCCAGTACTGTCAC	2865–2897
U-Mlu-1-BC	GGAAGTTCAATTAGGAATACCACACCCAGCAG	2813–2844
U-Mlu-2-BC	ACTGGATGTGGGGGACGCGTATTTTTCAGTTC	2873–2904
D-MluI-AE+BC	GTCCACGCGTGGTCTGAGGGATCTCTAGTTACCAG	9659–9689
Env-1	GCATTAGGGATCATTCACGCACAACAG	4053–4080
Env-3	CTGCTGTATTGCTACTCGTGATTGCTCCATG	8914–8944
Env-2	GAATCAGAGTTAGTTAACCAAATAATAG	4089–4116
Env-4	TTTTTCCAGGTCTCGAGATACTGCTCCCAC	8884–8913

**Figure 2:**
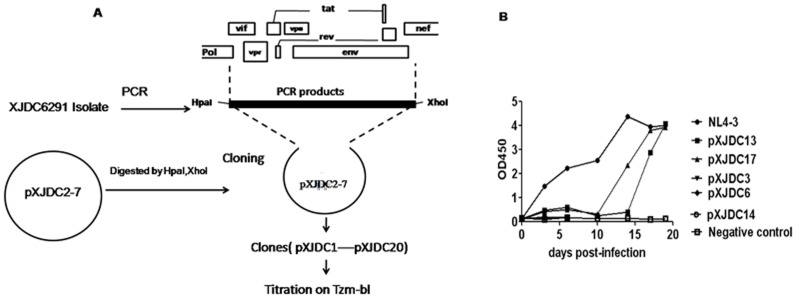
Repair of defective infectious clone pXJDC2-7. (A) Schematic diagram for repairing the defective infectious clone pXJDC2-7. The 4.6 kb fragment from the Integrase to the Nef gene was amplified from the XJDC6291 strain by PCR using unique restriction enzyme sites HpaI and XhoI. The corresponding gene fragment of the #2-7 clone was substituted by the bulk PCR products.(B) Replication capabilities of repaired clone-derived viruses in human PBMCs. Virus supernatant containing 1 ng p24 antigen was used to infect PHA-stimulated PBMCs respectively to test the replication capability of these viruses; NL4-3 strain was used as positive control. 1 ml supernatant was substituted by fresh medium every 3–4 days and strored at −80°C. Viral replication was measured by OD value in the supernatant at different time points with a p24 antigen-capture ELISA kit.

In order to replace the 4.8 kb cassette carrying the *vif*-*vpu*-*tat*-*rev*-*env*, partial *pol* integrase, and *nef* genes in the full-length HIV genomic clone, nested PCR was performed by using primers Env-1 and Env-3 for the 1st round PCR, Env-2 and Env-4 for the 2nd PCR with the Platinum Taq DNA polymerase High Fidelity kit (Table 1). PCR product was purified and used to take place of the corresponding fragment of full-length HIV clones by enzyme digestion of HpaI and XhoI(Fig. 2.A).

### Generation of Stock Virus

For the generation of clone-derived viruses, 0.8 µg full-length genome plasmid was transfected into 5×10^4^ 293T cells using the Lipofectamine 2000 (Invitrogen, USA) in 24 well plates, and supernatant was collected and 48 hours post infection, filtered through a 0.2 µm filter, and stored at −80°C for p24 quantification (BioMérieux, France), and TCID_50_ assays. TZM-bl cells were seeded in 24-well plates at a density of 10,000 cells/well in Dulbecco’s modified Eagle medium (DMEM) containing 10% fetal calf serum (FCS). Serial dilutions of 293T transfection-derived virus stocks were added in a final volume of 200 µl/well containing 40 µg/ml DEAE-dextran (Sigma,USA). Fourty-eight hours post-infection, plates were treated and Relative Light Unit (RLU) were measured.

### Determination of Virus Production by Electron Microscopy

Transfected cell pellets were fixed for electron microscopy (EM) with 2% paraformaldehyde and 2.5% glutaraldehyde in 0.1M sodium cacodylate buffer (PH7.2), postfixed after a buffer wash with 1% osmium tetroxide in the same buffer, dehydrated with a graded series of ethanol, and embedded in resin-Spurr. Ultrathin sections were cut on LKB ultratome and stained with uranyl acetate and lead citrate, and observed on FEI TECNAI 12 electron microscope with accelerating voltage of 80KV.

### Titrating Virus in Tzm-bl cells (TCID Assay)

100 µl of DMEM medium containing 10% FCS was placed in a 96-well flat-bottom culture plate. 25 µl of virus was used in the first 4 wells of a dilution series, and 25 µl was transferred to the next column to yield serial 5-fold dilutions for a total of 11 dilutions. Wells in column 12 served as cell controls for background luminescence (no virus added). 100 µl of TZM-bl cells (10,000 cells/100 µl DMEM containing 25 µg DEAE-Dextran/ml) were added into all wells and the plate was placed in a CO_2_ incubator for 48 hours. Following incubation, 100 µl of culture medium was removed from each well and 100 µl of Glo Lysis Buffer-Substrate mixture was dispensed to each well (Promega, USA) and incubated at room temperature for 2 minutes to allow complete cell lysis. 150 µl of lysate was transferd to a corresponding 96-well plate and read immediately in a luminometer (Perkin Elmer Life and Analytical Sciences). The TCID_50_ was calculated using the “TCID” assay software (provided by the Comprehensive Antibody-Vaccine Immune Monitoring Consortium, CAVIMC).

### Replication Capability of Infectious Clone Derived Virus

PBMCs were isolated from the peripheral blood of healthy HIV-1 seronegative donors using the Ficoll-Hypaque gradient method. The quantified TCID_50_ or p24 antigen HIV virus was used to infect 3×10^6^ phytohemaglutinin (PHA) -stimulated PBMCs in triplicate for two hours at 37°C in 1 ml total volume, following which the supernatant was removed and cells were washed by 10 ml PBS for three times. The cells were then cultured in 3 ml of completed RPMI 1640 medium (containing 10% FCS and 10 IU/ml IL-2) in a 25 cm^2^ flask (Corning, USA). Supernatant (1 ml) was substituted by fresh medium every 3–4 days, 3×10^6^ PHA -stimulated fresh PBMCs were seeded into the flasks every 7 days, and the supernatant obtained was filtered by a 0.2 µm filter and stored at −80°C.

To test the replication capability of virus in human primary CD4^+^ T cells, human CD4^+^ T cells were enriched by CD4 MicroBeads Kit(Miltenyi Biotec, Germany) from fresh human PBMCs. After cells were cultured in RPMI1640 supplemented with 10% FBS, 5 µg PHA/ml for 72 hours, 10^4^ TCID_50_ virus supernatant was added into 3×10^6^ PHA-stimulated CD4^+^ T cells in triplicate for two hours at 37°C in 1 ml total volume. The supernatant was then removed and cells were washed by 10 ml PBS for three times and cultured in 3 ml of completed RPMI1640 (containing 10% FCS and 10 IU/ml IL-2). The sample was collected every 3–4 days, and 3×10^6^ PHA -stimulated fresh CD4^+^ T cells were supplemented into the flasks every 7 days.

### Co-receptor Usage Analysis

We cultured 4×10^4^ Ghost-CCR5 and Ghost-CXCR4 cells in DMEM containing 10% FCS, 100 µg/ml of streptomycin, 100 IU/ml of penicillin, 500 µg/ml of geneticin,100 µg/ml of Hygromycin B and 50 µg/ml of puromycin in 24 well plates. We used reference HIV-1 molecular clones with known coreceptor usage, HIV-1 Bal for CCR5 and NL4-3 for CXCR4 as the positive control. To further confirm the coreceptor usage of the viruses, CCR5 antagonist TAK779 and CXCR4 antagonist AMD3100 were used to treat Ghost-CCR5 and Ghost-CXCR4 cells 1 hour prior of infection at the final concentration of 20 µM and 2.5 µM, separately. Virus of 800 TCID_50_ was used to infect the cells for 2 hours in triplicate, and then the supernatant was replaced by fresh medium. We removed the supernatant 48 hours post-infection and the cells were washed twice with 1 ml PBS, 200 ul 0.25% Trypsin/EDTA solution was added into the well and cultured at room temperature for 1 min. To stop the reaction, 200 ul Fetal Bovine Serum (FBS) were added into the well drop by drop, and approximately 4,000 cells each well were collected for flow cytometry assay to determine the number of GFP-expressing cells.

### P24 Antigen Detection

P24 antigen detection kit (Biomeriux, France) was used for quantitative or qualitative analysis of HIV-1 p24 antigen. For quantification of HIV P24, five dilution series of recombinant HIV-1 p24 antigen in the concentration range of 160 pg/mL −10 pg/mL and the serially diluted virus supernatant were performed ELISA test, absorbance of each microwell on a spectrophotometer using 450 nm wave length was measured. The standard regression line of p24 antigen-OD450 was prepared, and then the amount of p24 antigen in virus supernatant was calculated according to the standard regression line. For qualitative analysis of p24 antigen presence, the supernatant was measured without serial dilution and only OD450 value was measured.

### Sequence Analysis

The full-length sequences of the HIV-1 clones pXJDC2-7, pXJDC2-6, pXJDC3-6, pXJDC3-7, pXJDC13, and pXJDC17 were sequenced by primer walking and assembled using software Sequencher 4.10.1, and the genome sequence was submitted to the Gene Cutter tool (http://www.hiv.lanl.gov/content/sequence/GENE_CUTTER/cutter.html) in the Los Alamos National Laboratory HIV Database website (http://www.hiv.lanl.gov/content/index) to locate coding regions and generate nucleotide and protein alignments. Genome sequence recombination analysis was performed using the bootscanning function of the software Simplot (version 3.5).

### Statistics Analysis

Student’s t-test was applied to determine the statistical difference between two groups. All analyses were performed using a two-tailed t-test, and P values less than 0.05 were considered statistically significant.

## Results

### Construction of the Full-length HIV-1 CRF07_BC Clone Using the Two-partial Molecular Cloning Method

As shown in Fig. 1.A, a 2.9 kb fragment from the U3 region in the 5′LTR to the RT region, as well as a 6.9 kb fragment spanning the RT region to the U5 region in the 3′LTR, were amplified from the proviral DNA extracted from XJDC6291 infected PBMC. These two fragments were then separately subcloned into the low-copy plasmid pLG338 for sequencing. Two 5′partial subclones (#2 and #3) containing the 2.9 kb DNA fragment and two 3′ partial sublones (#6 and #7 ) carrying the 6.9 kb fragment were selected, with the MluI restriction site in the overlapping region of 5′ partial molecule and 3′ partial molecule. Using these subclones, four full-length recombinant clones #2–6 (pXJDC2-6), #2–7 (pXJDC2-7), #3–6 (pXJDC3-6), #3–7 (pXJDC3-7) were constructed with the very low copy vector pLG338. Sequence analysis indicated that all the four clones encoded a complete set of viral proteins and intact regulatory elements, including promoter and enhancer regions in the LTR, the trans-activating responsor (TAR) element and the packaging signal. No deadly stop codon or frame shift mutations were found in the four full-length genome clones.

### Production of Highly Infectious Viral Clones

After transfection of each infectious clone plasmid into 293T cells, the supernatants were collected 48 hours post-transfection to test for virus infectivity on Tzm-bl cells. As well, p24 antigen concentrations in the supernatant were quantified using antigen capture ELISA. All clones were positive for p24 antigen, but most of the supernatant from the clone-transfected cells displayed no infectivity, except the pXJDC2-7 clone which presented a higher TCID_50_ value of 112 TCID_50_/ml (Table 2).

**Table 2:** P24 antigen production and TCID_50_/ml of supernatant from plasmid transfected 293T cells.

Clone Number	0D_450_ Value (p24 antigen test)	P24 antigen quantification (ng/ml)	TCID50/ml
pNL4-3	3.901	36.78	15000
pXJDC3-6	3.816	28.31	<50
pXJDC3-7	3.66	11.98	<50
pXJDC2-6	4.033	16.56	<50
pXJDC2-7	4.146	26.67	112
pLG338	0.172	Not assessed	<50

To examine the replication capability of pXJDC2-7, 112 TCID_50_ clone-derived virus was propagated in PHA-stimulated PBMCs. The replication kinetics of pXJDC2-7 is shown in Figure 1B. The viral supernatant was unable to establish a productive infection as determined by the lack of evident increase in p24 antigen in the cellular supernatant over a 15-day period.

Weak infectivity of HIV virus particles may be due to many causes, including frameshift or stop codon mutations in the proviral genome. However, no major deletions, insertions, or arrangements were found in the reading frames or untranslated regions of the four produced molecular clones. At the same time, considering that defective particles account for a major proportion of the whole virus quasispecies [15], it is not unusual to have acquired replication-deficient genomic clones during molecular manipulation of plasma isolates. The HIV-1 envelope gene is the key factor in determining the infectivity and fitness of the virus. Ndung’u et al had constructed four clade C full-length clones, none of which was infectious even though these four clones expressed all the expected virion-associated proteins; however, they also found that the infectivity of two clones could be rescued by complementation with a functional subtype C envelope clone MOLE1 [16]. In order to determine whether Env deficiency caused the poor infectivity of our clone derived viruses, HIV-1 clade BC envelope plasmid CH120.6 was transfected with the equivalent clones of #2–7, #2–6, #3–6, #3–7 respectively. Infectivity of the resulting viruses was measured by RLU on Tzm-bl cells. Results showed that #2–7 co-transfected with the CH120.6 Env plasmid can increase the infectivity of the clone derived virus by ten times (P<0.0001), and the infectivity of viruses derived from the other two - clones (#3–6 and #3–7) co-transfeted with CH120.6 Env also increased (Figure 1C). This result suggests that there may be functional defects with the Env gene of clone #2–7.

To ameliorate the defective fitness of the #2–7 derived virus, the 4.6 kb fragment from the Integrase to the Nef gene was amplified from the XJDC6291 primary isolate, and the corresponding gene fragment of the #2–7 clone was replaced by the bulk PCR products using the unique restriction sites HpaI and XhoI (Fig. 2.A). Twenty clones were selected for transfection. 48 hours after transfection, viral supernatant was collected to perform TCID_50_ assay on Tzm-bl cells. Five clone-derived viruses displayed relatively high TCID_50_, from 559 to 6250 (Table 3). Consequently, virus stocks containing 1 ng p24 antigen were used to infect PBMCs separately to test the replicative capability of these viruses. Figure 2B shows that the pXJDC13 and pXJDC17 derived viruses could replicate in the PBMCs but others could not. After 20 days of culturing, p24 antigen content in the supernatant of pXJDC-13 and pXJDC-17 infected cells reached 10.76 ng/ml and 7.34 ng/ml, and the TCID_50_ tested in the Tzm-bl cells was 69877 TCID_50_/ml and 10687 TCID_50_/ml (Table 3). These data indicated that the two clones have stronger replication capability in vitro.

**Table 3:** Infectivity and replication capability of repaired viruses.

Clone Number	Infectivity of viruses (TCID_50_/ml)	20 days post infection on PBMCs	
		P24 (ng/ml)	TCID_50_/ml
pXJDC13	6250	10.76	69877
pXJDC17	6250	7.34	10687
pXJDC3	559	Negative	Not assessed
pXJDC6	559	Negative	Not assessed
pXJDC14	559	Negative	Not assessed
pNL4-3	13975	31.15	Not assessed

### Biological Characteristics of Infectious Clone pXJDC13 and pXJDC17

The transfected 293T cells were collected for electron microscopy. In the pXJDC-13 transfected cells, a typical HIV virus particle showing the conical condensed core and thin envelope was observed (Figure S1A). Interestingly, in this sample, there were also some particles with ring shaped core and thick envelope (Figure S1B). These are the immature HIV particles in which Pr55^Gag^ was not cleaved into the MA (Matrix), CA (Capsid), and NC (Nucleocapsid) structural proteins by protease [17]. In the pXJDC-17 transfected cells, different shapes of HIV virus particles were found containing an amorphous or non-conical core as well as a conical core (Figure S1C). The budding process was also observed clearly (Figure S1D). Thus, the constructed full-length molecular clones could generate functional virus particles.

**Figure 3:**
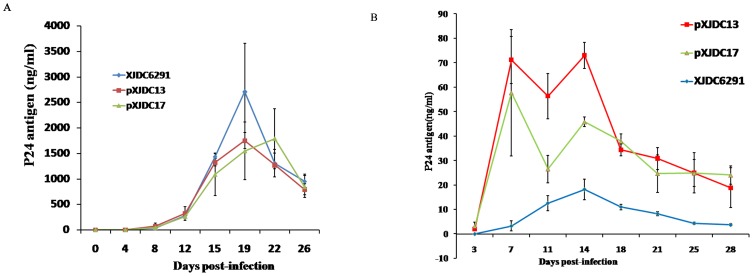
Replication kinetics of the viruses derived from the infectious molecular clone in comparison with parental isolate XJDC6291. 3×10^6^ PHA-stimulated PBMCs or CD4^+^ T cells were infected with virus normalized at a TCID_50_ of 10^4^ in triplicate. Virus production was monitored by measuring HIV-1 p24 antigen concentration in culture supernatant every 2–3 days. The results shown are the mean value with standard deviations from three independent experiments. (A) Replication kinetics on PBMCs. (B) Replication kinetics on CD4^+^ T cells.

The XJDC6291 primary isolate, pXJDC-13 and pXJDC-17 derived viruses were used to infect PHA-stimulated PBMCs with the 10^4^TCID_50_ dosage. After 4 days, supernatant from infected cells were positive for p24 antigen; after 22 days, p24 antigen level reached more than 1500 ng/ml (Figure 3A). The p24 antigen peak level is much higher than those reported in other publications regarding BC infectious clones pBRNG and NLXJDC6441X2, which were less than 100 ng/ml and 20 pg/ml respectively [10], [13].

To further characterize the biological property of the cloned viruses, the replication levels of the cloned viruses were also tested in human primary CD4+ T cells. pXJDC13 and pXJDC17-derived viruses presented higher replication levels than the parental XJDC6291 primary isolate. pXJDC13-derived virus reached p24 peaks over 70 ng/ml on day 7 and day 14 post-infection, and pXJDC17-derived virus had peak p24 level exceeding 40 ng/ml at these two time points, while peak p24 level for the parental isolate XJDC6291 was only 15 ng/ml, on day 14 (Figure 3.B).

**Figure 4:**
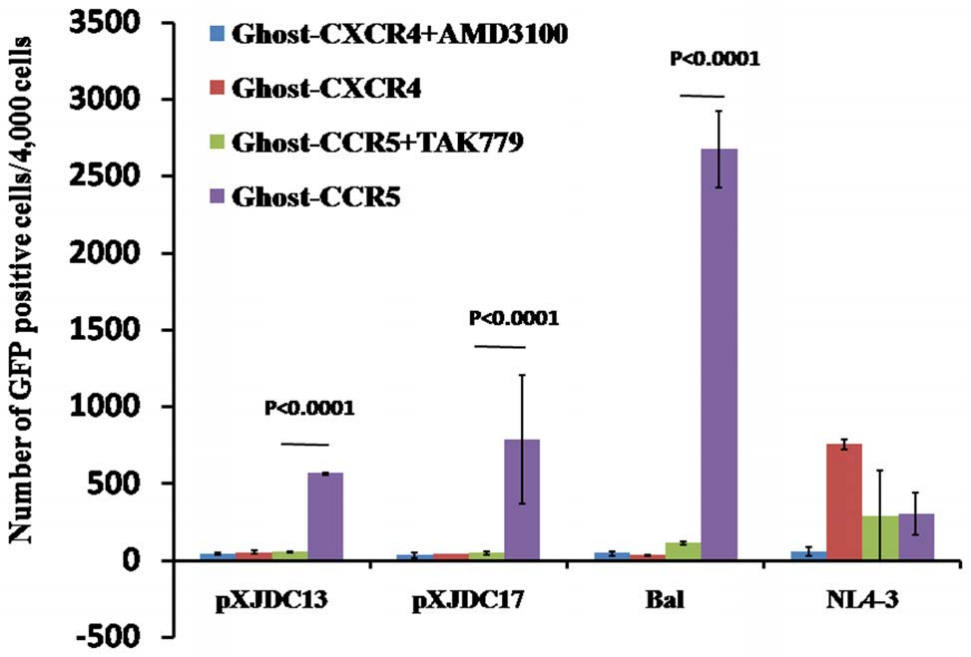
Tropism of clone derived viruses and their parental isolate. Ghost-CXCR4 and Ghost-CCR5 cells were treated in the absence or presence of the following inhibitors one hour prior to infection: CXCR4 antagonist AMD3100 at 2.5 µM, or CCR5 antagonist TAK-779 at 20 µM. Approximately 4,000 cells for each sample were collected to perform flow cytometry assay for GFP signals to determine the number of HIV infected cells. The mean numbers of GFP-positive cells are shown with standard deviations from three separate assays.

Finally, Ghost-CCR5 and Ghost-CXCR4 cells were infected by our virus stock containing 800 TCID_50_ in triplicate. We analyzed the infected Ghost cells by flow cytometry 48 hours post-infection. Both infectious clones and their parental isolate infected the Ghost-CCR5 cells at a significantly higher level than in the Ghost-CXCR4 cells, and could be inhibited by the CCR5 antagonist TAK779 (Figure 4). The clones exhibited exclusive CCR5 tropism, consistent with most CRF07_BC viral isolates reported in the literature [10], [13], [14].

### Sequence Signatures of the Full-length Molecular Clones

The completed nucleotide sequences of the molecular clones were submitted to Genbank. The accession numbers of pXJDC13 and pXJDC17 are KC492737 and KC492738 respectively, and pXJDC2-6, pXJDC2-7, pXJDC3-6, pXJDC3-7 are available in the GenBank under the accession numbers KC503852-KC503855.

Bootscan analysis by reference isolates from each subtype did not show any new breakpoints in the pXJDC13 sequence, and also confirmed that XJDC6291 (Figure S2 A) has a C/B_ mosaic structure, similar to that of typical CRF07_BC strain CN54 (Figure S2 B). Consistent with the original analysis, the majority of the pXJDC6291-13 infectious molecular clone sequence was determined as subtype C, while the internal portion of its *gag, pol, env,* and *nef* and the first exon of *tat* was presumed to have originated from subtype B (Thai-B).

The LTR region is very critical to HIV transcription. Normally, HIV clade BC or C own two or three promoters in the LTR, and XJDC6291 also contained a C clade specific NF-κB site except for the other two promoters NF-κB (II) and NF-κB (I) (Figure S3 A). The truncated *rev* gene is also found in the XJDC6291 infectious clone, in which a premature stop codon caused a 16 amino acid truncation (Figure S3 B). However, *rev* gene truncations are also found in some clade C or BC strains, hence the premature stop codon in this position would not affect the function of Rev.

We compared the amino acid sequences of pXJDC13 and pXJDC17 with those found in the clone pXJDC2-7. Point mutations R51H and G54D were found within the p31 integrase region in both clones (Figure S4 A). Given that arginine (R) and histidine (H) are both positively charged amino acids and that glycine (G) and aspartic acid (D) are both negatively charged, these two mutations may not have affected the function of integrase greatly. As well, an S59P mutation was found in the Tat gene in pXJDC13 and pXJDC17 (Figure S4 B). Considering that the pXJDC2-7 derived virus can also activate the Tat-responsive luciferase gene in the Tzm-bl cells, the mutation is not responsible for recovering the virus replication capability. Two other mutations were also identified in the Nef gene (Figure S4 C): A32T in pXJDC13 and C206Y in pXJDC17, respectively, but appeared to have no relation to replication capability, as was the case for additional mutations in the Vpu gene (Figure S4 D).

**Figure 5:**
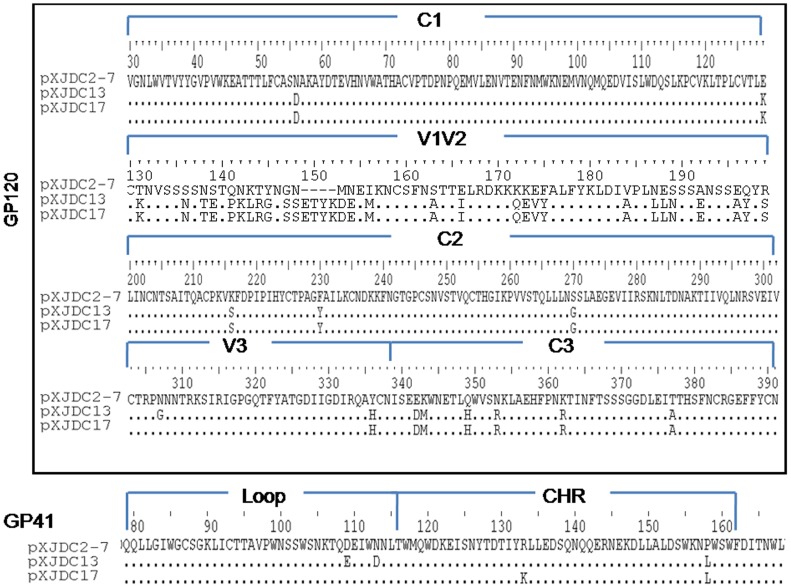
Differences in amino acid sequence in the *env* gene between the two infectious clones (pXJDC13, pXJDC17) and replication-defective clone pXJDC2-7. Substitutions or deletions within each region are shown; conserved amino acids are indicated with the character “.” and deletions are indicated with the character “−”.

On the other hand, multiple mutations were identified among the *env* sequences of the three clones in C1, V1V2, C2, V3, and C3 of gp120 and the loop region CHR within gp41 (Figure 5). In particular, the V1V2 region of pXJDC13 and pXJDC17 contained 70 amino acids while that of pXJDC2-7 contained 66 amino acids, with point polymorphisms. It is most likely that the substitutions and insertions here are associated with the higher replication capability of pXJDC13 and pXJDC17 derived viruses; further investigation is necessary.

### Impact of V1V2 Region Characteristics on HIV Virion Infectivity and Replication Capability

**Figure 6:**
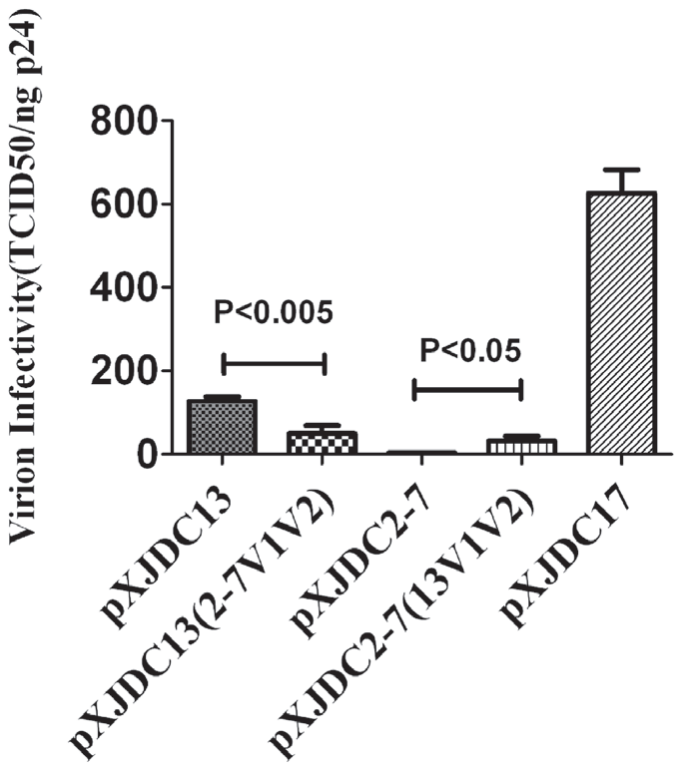
Measurement of infectivity of clone-derived viruses. The clone-derived virus was produced by transfection into 293T cells and stored at −80°C. Frozen virus supernatant was then thawed to test the TCID_50_ value on Tzm-bl cells and p24 antigen levels. Virion infectivity was calculated by TCID_50_/ng p24. The mean values are shown with standard deviations from three separate assays.

The V1V2 region of pXJDC2-7 and pXJDC13 was exchanged for each other by unique enzyme sites BlpI and ClaI (base 4727-7106 in XJDC6291 genome), which produced two chimeric clones pXJDC2-7 (13V1V2) and pXJDC13 (2-7V1V2). The resulting infectious clone-derived viruses were assayed for TCID_50_ and p24 antigen concentration, and the infectivity of chimeric viruses was shown as the index of TCID_50_/ng p24. Thus, V1V2 from pXJDC13 was shown to significantly improve the infectivity of chimeric virus pXJDC2-7(13V1V2), while V1V2 from pXJDC2-7 decreased the infectivity of chimeric virus pXJDC13(2-7V1V2)(Figure 6).

**Figure 7:**
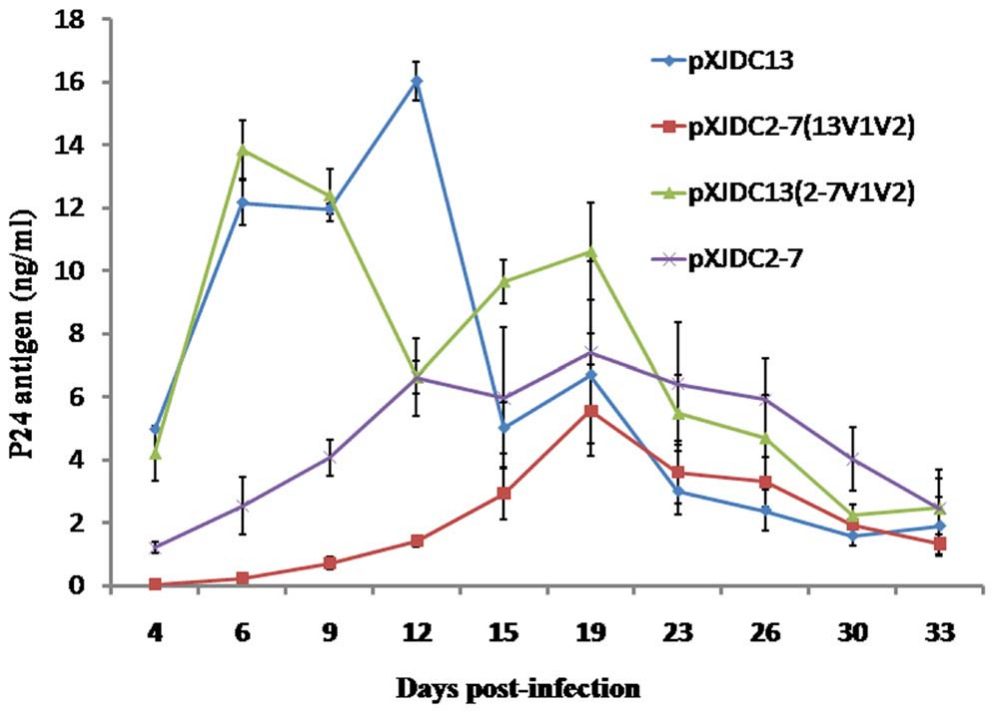
Replication kinetics of the chimeric viruses derived from the molecular clones pXJDC13(2-7V1V2) and pXJDC2-7(13V1V2), in comparison with parental clones pXJDC13 and pXJDC2-7. 3×10^6^ PHA-stimulated PBMCs were infected with viruses normalized at 400 TCID_50_ in triplicate. Virus production was monitored by measuring HIV-1 p24 antigen concentration in culture supernatant every 3–4 days. The results shown are the mean value with standard deviations from three independent experiments.

Finally, equal amounts of infectious clone-derived viruses pXJDC2-7, pXJDC13, and their chimerical derivatives (400 TCID_50_) were inoculated into the PBMCs. After 33 days culture, all four clones could replicate in the PBMCs, but the two chimerical viruses did not show the obvious variation compared to their parental viruses (Figure 7).

## Discussion

In this study, we constructed highly infectious full-length HIV-1 CRF07_BC molecular clones that can replicate efficiently in human PBMCs and that use CCR5 as the entry co-receptor. We observed differently shaped HIV particles and viral budding of this HIV-1 CRF07_BC molecular clone through electron microscopy. These infectious clones will provide a unique tool to study the HIV CRF07_BC virus characteristics.

To date, several infectious molecular clones from isolates of different subtypes have been constructed, but acquiring a molecular clone with similar replication capability to the primary isolate remains a challenge. Previously, we reported a molecular clone of CRF07_BC [13]. It is a chimeric clone with the complete 5′ LTR, 3′LTR and partial nef gene from NL4-3 infectious clone, and that clone exhibited very weak replicable capability in PBMCs. Mutations in the genome or polymorphisms in the *rev*-*vpu*-*env* cassette often resulted in replication-defective virus [18], but sequencing results are typically unable to pinpoint the exact cause. In the construction of XJDC6291 derived strains, four full-length molecular clones pXJDC2-6, pXJDC2-7, pXJDC3-6, pXJDC3-7 were initially produced, but only pXJDC2-7 was able to produce infectious virus and replication efficiency in PBMCs was low. However, its infectivity was rescued by substitution of a large fragment covering partial *pol* gene, whole *vif*-*vpr*-*tat*-*vpu*-*rev*-*env* region, and partial *nef* gene from the XJDC6291 isolate. Our analysis of polymorphisms revealed that the V1V2 region was most likely responsible for this effect. The V2 gene region is responsible for binding of the α4β7 integrin on the surface of CD4^+^ T cells of the mucosa-associated lymphoid tissue (MALT) [19], and previous studies had suggested that V1V2 may play an important role in HIV replication [20]. In comparing the *env* sequence of pXJDC2-7 with pXJDC13 and pXJDC17, it is difficult to infer which mutations are critical to rescuing the viral infectivity; as well, exchanging the whole V1V2 region improved infectivity but did not increase replication capability. Further investigation is needed to clarify the roles of the observed polymorphisms.

Regarding the replication capability of previously constructed CRF_BC infectious clones, the chimeric CRF07_BC NLXJDC6441X2 infectious clone only has a peak p24 antigen less than 20 pg/ml and the CRF08_BC infectious clone pBRNG has a peak p24 antigen concentration below 100 ng/ml. In comparison, the pXJDC13 and pXJDC17 could display a higher peak p24 antigen over 1500 ng/ml, much higher than the previous reported infectious C/BC clade clones [10], [13], [21].

In summary, as the first highly replication-competent infectious molecular clone of HIV-1 subtype CRF07_BC, a subtype that dominates in China, pXJDC13 and pXJDC17 constitute an important tool that can be used for HIV research in China. The two clones were confirmed to be highly replication competent in PBMCs and were observed to be CCR5 tropic. The availability of these infectious clones will facilitate the study of virulence and recombination mechanisms of subtype-B/C CRFs circulating in China. Furthermore, the swapping of large genomic fragments containing the *env* region used in the study may be a convenient way to rescue a replication-defective HIV clone.

## Supporting Information

Figure S1 **Morphology of clone-derived virus particles after transfection of 293T cells, observed with transparent electron microscopy.** (A) Electron micrograph showing the pXJDC13 derived virus budding from the 293T cells. (B) Premature virus particles of pXJDC13 clone derived virus. (C) Mature virus particles of pXJDC17 clone. (D) pXJDC17 derived virus is budding from the transfected 293 T cells. Scale is shown by a size bar.
(TIF)

Figure S2 (A) Bootscan analysis of isolate XJDC6291 queried against three reference sequences B′CN.RL42, C.IN.1993. 93IN101, and 01_AE.93JP_NH1. Bootscan analysis was performed using SimPlot 3.5.1 software with 1000 bootstrap replicates, 1000 bp window and a step size of 50 bp. The x-axis shows all aligned nucleotides of the sequence analyzed and the y-axis shows the bootstrap value. (B) The mosaic structure of CRF07_BC XJDC6291. Breakpoints were determined from two sequences of B′CN.RL42 and C.IN.1993. 93IN101 using the jpHMM program. The schematic structure was created using the Recombinant HIV-1 Drawing Tool. Both programs are available on the Los Alamos HIV sequence Database website (http://www.hiv.lanl.gov/content/sequence/HIV/HIVTools.html).
(TIF)

Figure S3 (A) Alignment of promoters in the LTR between pXJDC6291-13 and HIV-1 clade B and clade BC reference strains. (B) Alignment of *rev* gene between pXJDC6291-13, pXJDC6291-17 and HIV-1 clade B and clade BC reference strains.
(TIF)

Figure S4 **Alignment of amino acid sequences between pXJDC6291-13, pXJDC6291-17 and pXJDC6291-2-7.** The segments are: (A) integrase, (B) tat, (C) nef, and (D) vpu.
(TIF)
