# Supplementary figures and images for: Construction and Characterization of Highly Infectious Full-Length Molecular Clones of a HIV-1 CRF07_BC Isolate from Xinjiang, China
Source: PLoS One. 2013 Nov 18;8(11):e79177. doi: 10.1371/journal.pone.0079177 (PMC3850540; doi:10.1371/journal.pone.0079177)

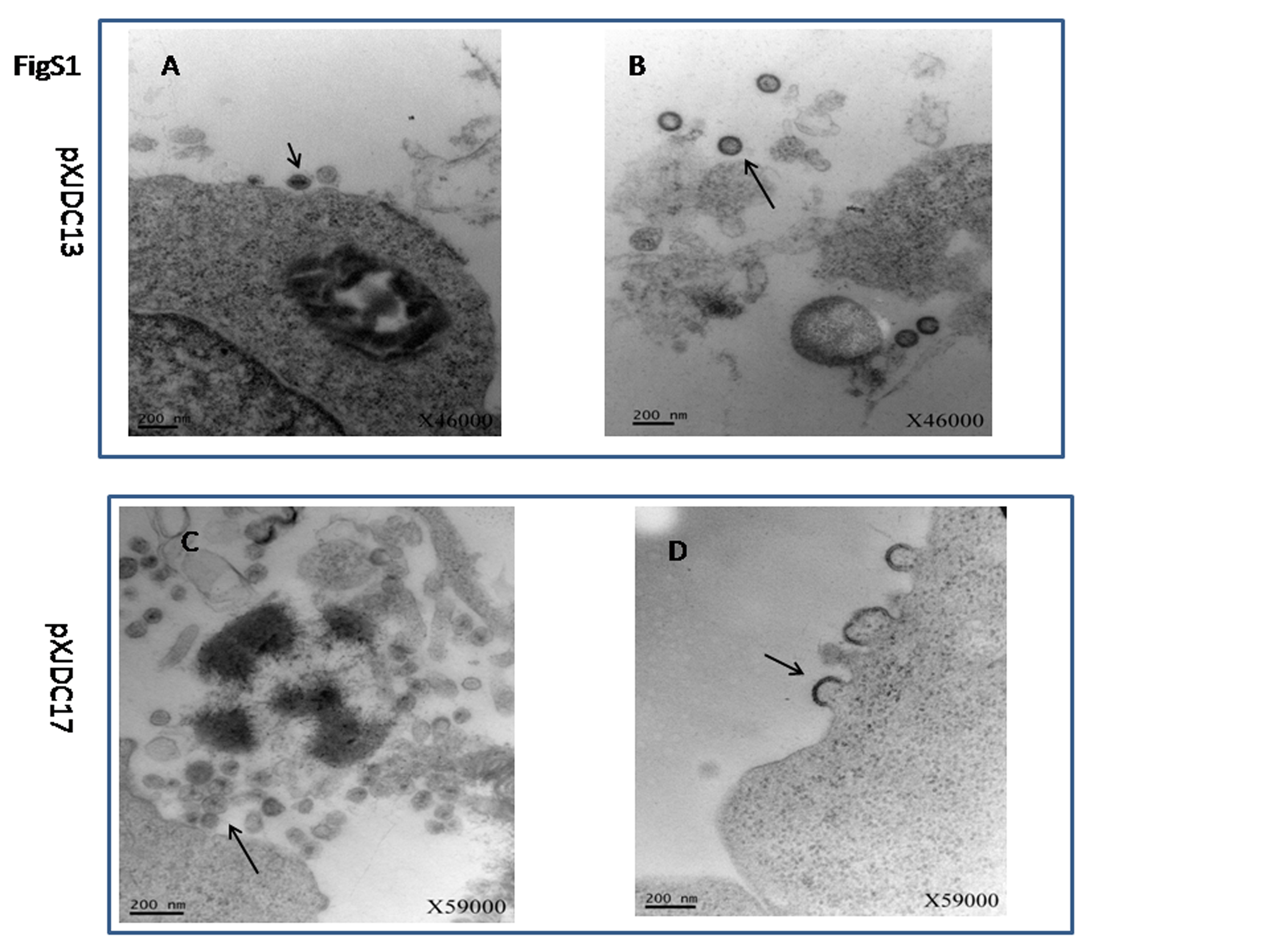

Supplement: Figure S1 — Morphology of clone-derived virus particles after transfection of 293T cells, observed with transparent electron microscopy. (A) Electron micrograph showing the pXJDC13 derived virus budding from the 293T cells. (B) Premature virus particles of pXJDC13 clone derived virus. (C) Mature virus particles of pXJDC17 clone. (D) pXJDC17 derived virus is budding from the transfected 293 T cells. Scale is shown by a size bar. (TIF) [file pone.0079177.s001.tif]

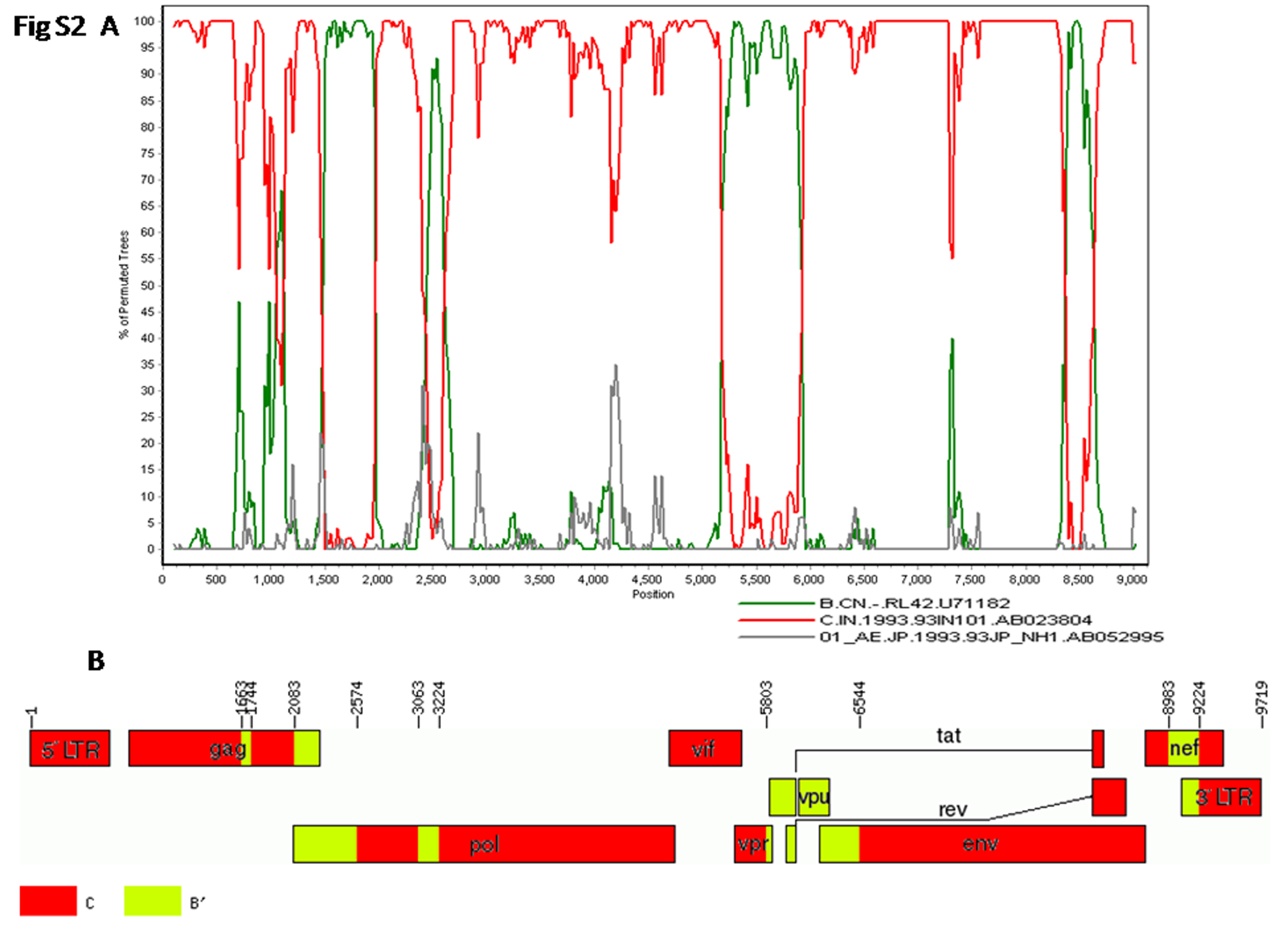

Supplement: Figure S2 — (A) Bootscan analysis of isolate XJDC6291 queried against three reference sequences B′CN.RL42, C.IN.1993. 93IN101, and 01_AE.93JP_NH1. Bootscan analysis was performed using SimPlot 3.5.1 software with 1000 bootstrap replicates, 1000 bp window and a step size of 50 bp. The x-axis shows all aligned nucleotides of the sequence analyzed and the y-axis shows the bootstrap value. (B) The mosaic structure of CRF07_BC XJDC6291. Breakpoints were determined from two sequences of B′CN.RL42 and C.IN.1993. 93IN101 using the jpHMM program. The schematic structure was created using the Recombinant HIV-1 Drawing Tool. Both programs are available on the Los Alamos HIV sequence Database website (http://www.hiv.lanl.gov/content/sequence/HIV/HIVTools.html). (TIF) [file pone.0079177.s002.tif]

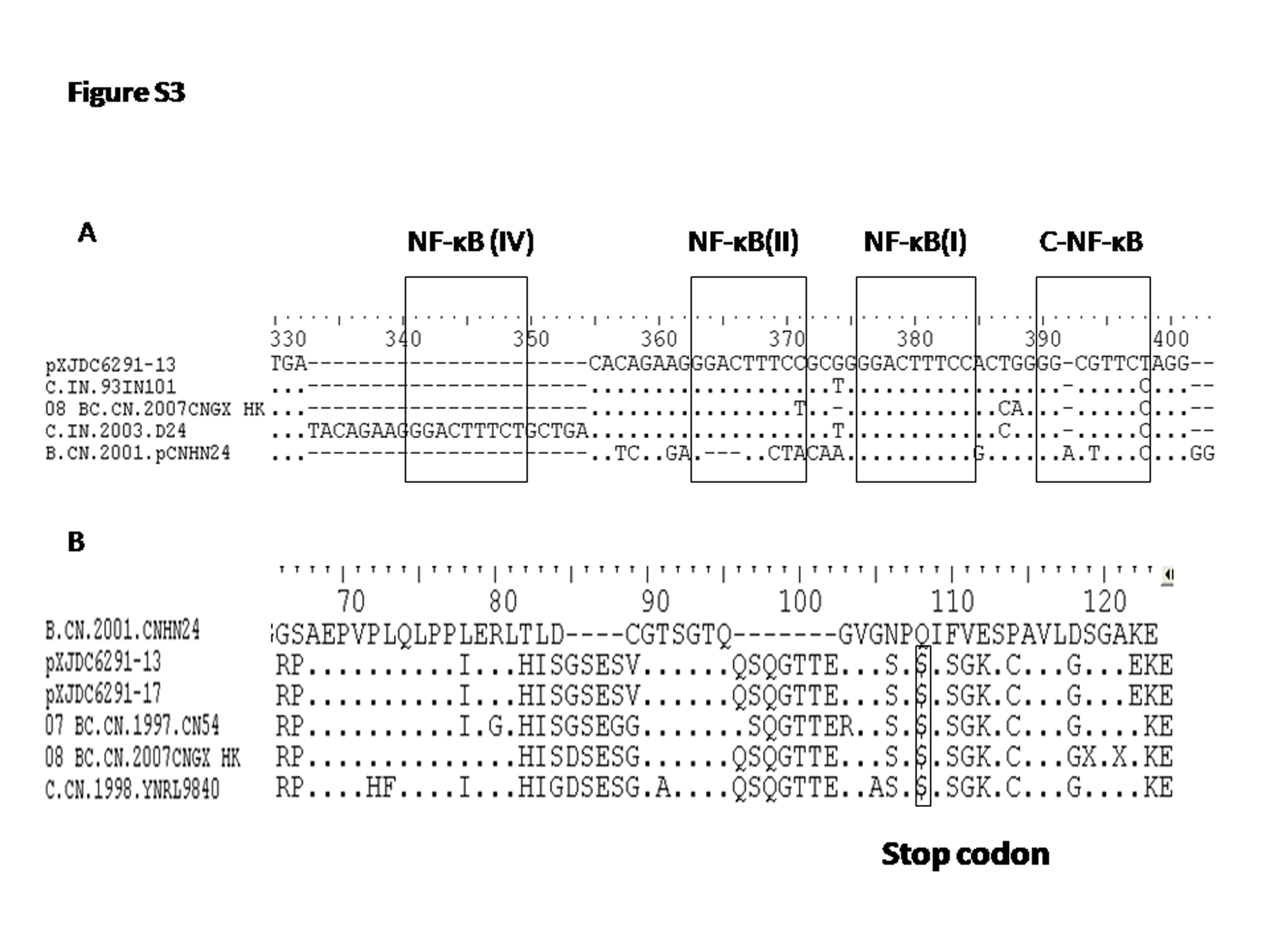

Supplement: Figure S3 — (A) Alignment of promoters in the LTR between pXJDC6291-13 and HIV-1 clade B and clade BC reference strains. (B) Alignment of rev gene between pXJDC6291-13, pXJDC6291-17 and HIV-1 clade B and clade BC reference strains. (TIF) [file pone.0079177.s003.tif]

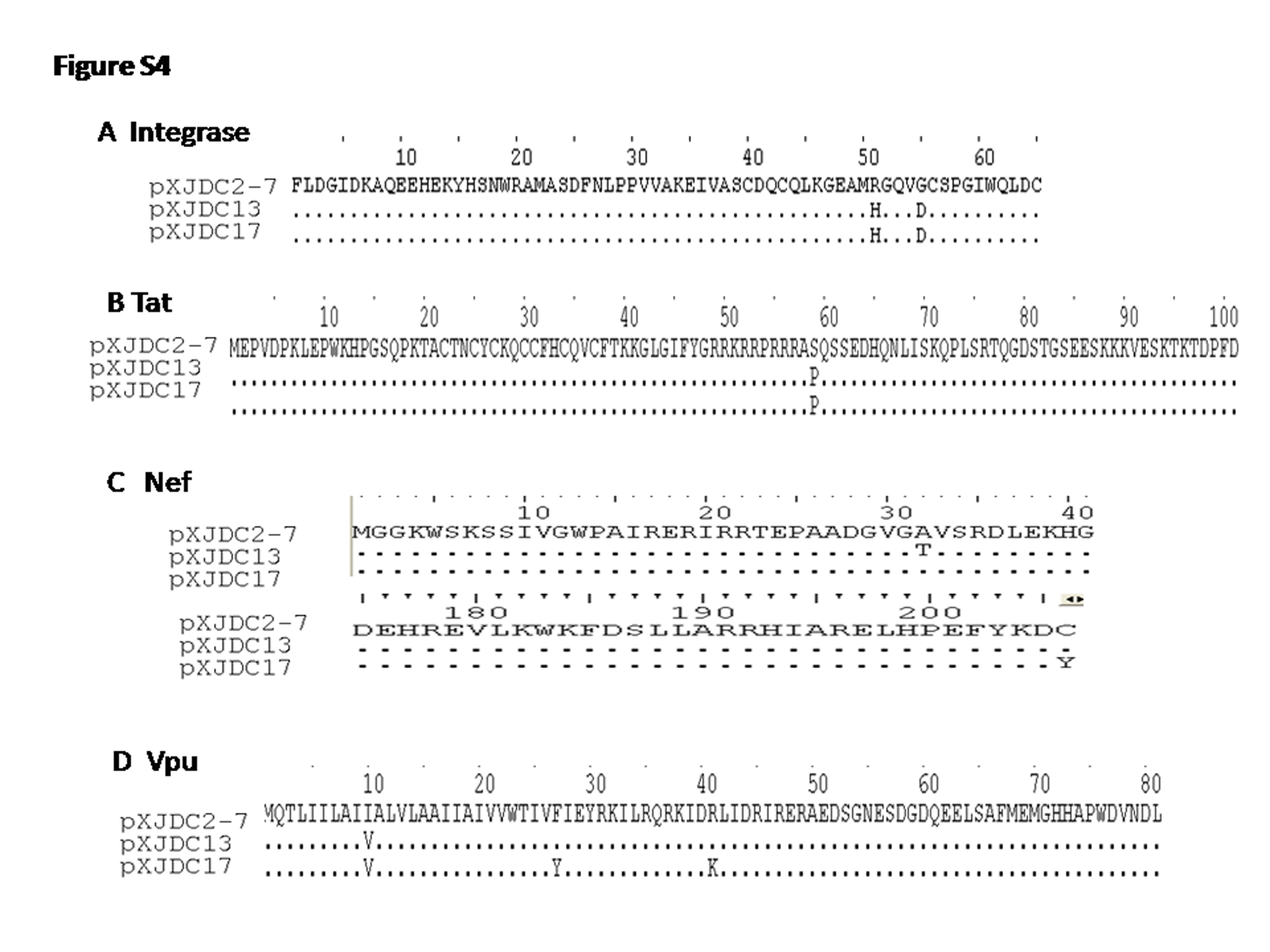

Supplement: Figure S4 — Alignment of amino acid sequences between pXJDC6291-13, pXJDC6291-17 and pXJDC6291-2-7. The segments are: (A) integrase, (B) tat, (C) nef, and (D) vpu. (TIF) [file pone.0079177.s004.tif]
